# Enhanced film thickness for Néel wall in soft magnetic film by introducing strong magnetocrystalline anisotropy

**DOI:** 10.1038/srep20140

**Published:** 2016-01-29

**Authors:** Fei Xu, Tao Wang, Tianyong Ma, Ying Wang, Shimeng Zhu, Fashen Li

**Affiliations:** 1 Key Laboratory for Magnetism and Magnetic Materials of the Ministry of Education, Lanzhou University, Lanzhou 730000, China

## Abstract

This study investigated the magnetic domain walls in a single-layer soft magnetic film with strong magnetocrystalline anisotropy energy. The soft magnetic film is composed of a highly c-axis-oriented hcp-Co_81_Ir_19_ alloy with strong negative magnetocrystalline anisotropy. The domain structure of the soft Co_81_Ir_19_ films with thickness ranging from 50–230 nm in a demagnetization state was observed through magnetic force microscopy and Lorentz transmission electron microscopy. Results reveal that the critical transition thickness at which the domain wall changes from Néel type to Bloch type is about 138 nm, which is much larger than the critical value of traditional Fe- and Co-based soft magnetic films with negligible magnetocrystalline anisotropy. Theoretical calculation was also performed and the calculated result agrees well with experimental data.

Magnetic domains and domain walls are major features in magnetism^1^. The microstructure of a magnetic domain and a domain wall type are important factors that affect material’s properties and determine material’s applications. For instance, soft magnetic thin films exhibit a wide range of applications, such as perpendicular magnetic recording, magnetic sensors, high-frequency inductor devices, miniature sensor, noise suppressor, and spin valve devices. The domain structure and domain walls play important roles in their applications. In perpendicular magnetic recording, the existence of Bloch wall in a soft magnetic underlayer causes the appearance of an out-of-plane stray field. This feature can leads to an additional noise signal during information reading^2^,^3^. In microwave assisted magnetic recording, the magnetization of a field generation layer inside a spin torque oscillator needs to be strictly aligned in the plane to stabilize magnetic rotation in the field generation layer^4^. In high-frequency applications, the appearance of stripe domains, in which most magnetic moments are not parallel to the film plane, directly affects the high-frequency magnetic properties when film thickness is too large^5^,^6^,^7^,^8^. Therefore, making sure that the magnetic moments strictly lie in the plane and maintaining the Néel wall in thicker soft magnetic films are important for present and potential applications. To accomplish these initiatives, researchers contributed greatly in this field. Néel wall can be formed in thicker soft magnetic films through several promising method, such as construction of multilayer structure films by inserting nonmagnetic insulating phase^9^,^10^,^11^,^12^ or introduction of antiferromagnetic coupling^13^,^14^,^15^,^16^ in Fe- or Co-based magnetic thin films. However, the construction of multilayer structure film is somewhat cumbersome and complex. Therefore, it would be more innovative and convenient if the Néel wall in a thicker layer can be achieved by changing the intrinsic parameters of the magnetic material^17^.

In soft magnetic films, the type of domain wall depends on domain wall energy. Here we focus on the 180° domain wall. The basic types of domain wall are Néel and Bloch walls, whose domain wall energies are expressed as follows^17^:


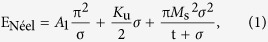



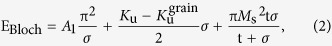


where t, *σ*, *K*_u_, 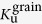, *M*_s_, and *A*_1_ denote magnetic layer thickness, domain-wall thickness, in-plane uniaxial anisotropy energy, magnetocrystalline anisotropy energy, saturation magnetization of the film and exchange stiffness constant, respectively. The type of domain wall depends on which is smaller for 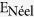 and 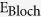. In most of the reported soft magnetic films, magnetocrystalline anisotropy energy (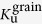) can be neglected. The domain wall assumes the form of Néel wall (the magnetic moments strictly lie in the film plane in the domain wall district) when the film is thin and it will become Bloch wall (an out-of-plane stray field exists in the domain wall district) when the film thickness exceeds a critical value. In conventional Fe- and Co-based soft magnetic films, this critical value is approximately 20–40 nm^18^,^19^,^20^,^21^,^22^. If the magnetocrystalline anisotropy energy is introduced to the soft magnetic film, the domain wall energy of Bloch wall expressed in eq. (2) increases; conversely, the energy of Néel wall remains unchanged. In this case, the critical thickness from Néel wall to Bloch wall can be enhanced.

Hcp Co_1−x_Ir_x_ (x ~ 0.2) soft magnetic material has a strong negative magnetocrystalline anisotropy^17^,^23^,^24^. In this situation, the c crystal plane is an easy magnetization plane, and c axis is a hard axis. If a film composed of the hcp CoIr grains growing with the c crystal plane parallel to the film plane is prepared, the soft magnetic film with strong magnetocrystalline anisotropy energy can be achieved. This film possesses soft magnetic properties in film plane. However, the rotation of magnetic moments to a perpendicular direction from the film plane must exceed the strong magnetocrystalline anisotropy energy besides the demagnetization energy. Although the enhanced film thickness for Néel wall in soft magnetic film with strong negative magnetocrystalline anisotropy has been predicted in theory^17^, the direct observation in experiment is not performed until now. In the present work, the critical transition thickness from Néel wall to Bloch wall was thoroughly investigated in the oriented Co_81_Ir_19_ soft magnetic film through experimental observations.

## Results and Discussion

**Figure 1:**
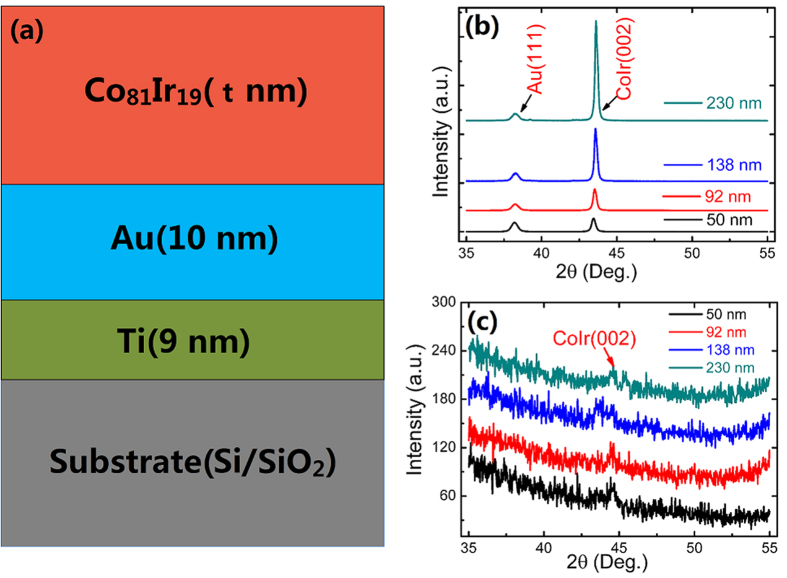
Layer structure and XRD pattern of the oriented Co_81_Ir_19_ films with thickness of 50 nm, 92 nm, 138 nm, and 230 nm.

Figure 1a shows the layer structure of the prepared films various Co_81_Ir_19_ thicknesses (t = 50, 92, 138, 230 nm). Only two diffraction peaks are observed in all the films: the peak of (111) and (002) plane for Au and Co_81_Ir_19_ in the mode of θ–2θ scan, respectively (Fig. 1b). When the samples are characterized through grazing incidence XRD, the patterns shown in Fig. 1c reveal only a very weak peak of (002) crystal plane, and no other peaks are observed. Therefore, the highly c-axis-oriented hcp-Co_81_Ir_19_ films are successfully prepared on the underlayer of (111)-oriented Au.

**Figure 2:**
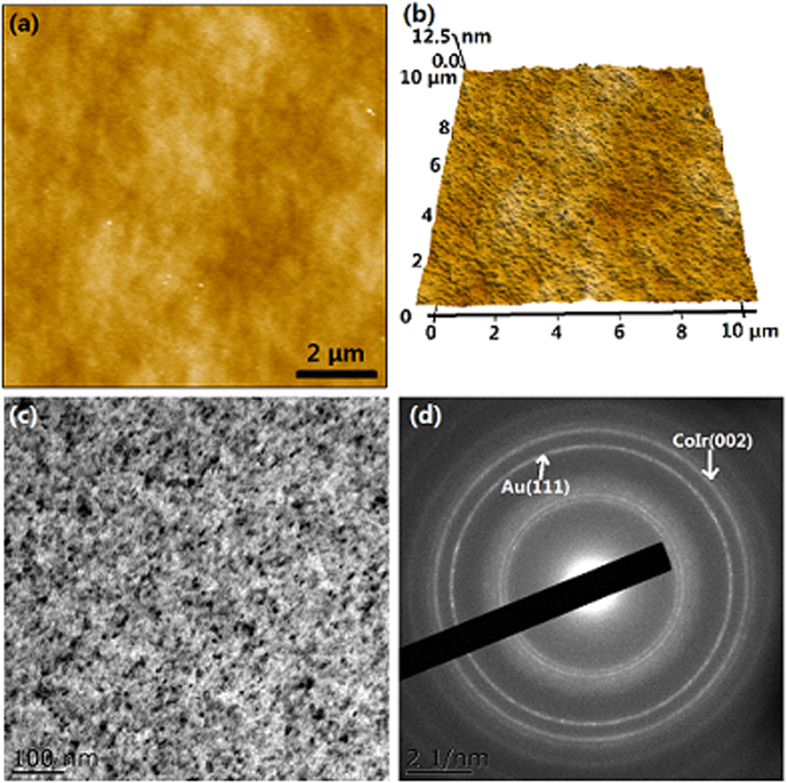
Microstructure of Co_81_Ir_19_ film with t = 50 nm. (**a**) AFM image; (**b**) AFM 3D-morphology; (**c**) HRTEM pattern; (**d**) SAED pattern.

Figure 2 displays the microstructure of the Co_81_Ir_19_ film with thickness of 50 nm. Figure 2a is the atomic force microscopy (AFM) topography of the film with the size of 10 μm × 10 μm. Figure 2b shows the 3D morphology of the film, which confirms that the film is uniform and continuous. The root-mean-square roughness (RMS) of the film is 8.0 Å. Figure 2c shows the transmission electron microscopy (TEM) topography of the film in the scale of 100 nm. The TEM topography also confirms that the specimen is uniform and continuous. The detailed crystalline information of the films is shown in Fig. 2d. The selected area electron diffraction pattern reveals that the film displays a polycrystalline structure. The diffraction rings correspond to (111) plane of Au and (002) plane of CoIr. This observation is strong evidence that the Co_81_Ir_19_ grains grow with the c crystal plane parallel to the film plane.

**Figure 3:**
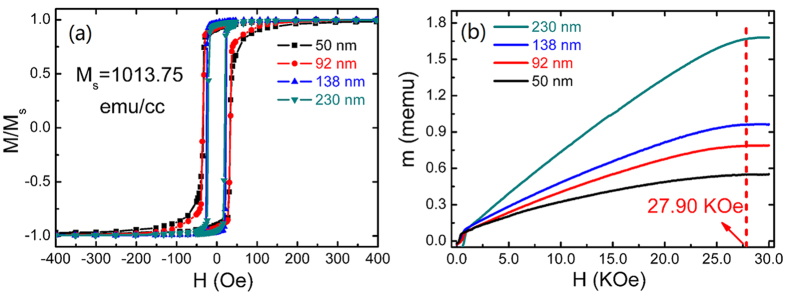
Static magnetic properties of Co_81_Ir_19_ film with t = 50 nm, 92 nm, 138 nm, 230 nm. (**a**) In-plane hysteresis loops of Co_81_Ir_19_ film with the applied magnetic field parallel to the film plane. (**b**) Initial magnetization curve of Co81Ir19 film with the applied magnetic field perpendicular to the film plane.

The in-plane magnetic hysteresis loops with the applied field along the easy axis of the films with different thicknesses have been measured, and the results are shown in Fig. 3a. The films exhibit good soft magnetic properties. The coercivity of the films gradually reduces from 33.5 Oe to 20.5 Oe as the Co_81_Ir_19_ layer thickness increases. This variation tendency is consistent with the previous report^25^. Figure 3b shows the out-of-plane initial magnetization curves for the samples with different thicknesses. The required saturation fields are nearly the same for all the films, ~27.90 kOe. Considering that the demagnetizing field of the Co_81_Ir_19_ soft magnetic films is only 12.74 KOe, the much larger saturation field comes from the strong negative magnetocrystalline anisotropy of Co_81_Ir_19_ grains. The nearly similar saturation field of the films with different thicknesses suggests that the orientation degree of the Co_81_Ir_19_ film is not deteriorated as film thickness increases.

**Figure 4:**
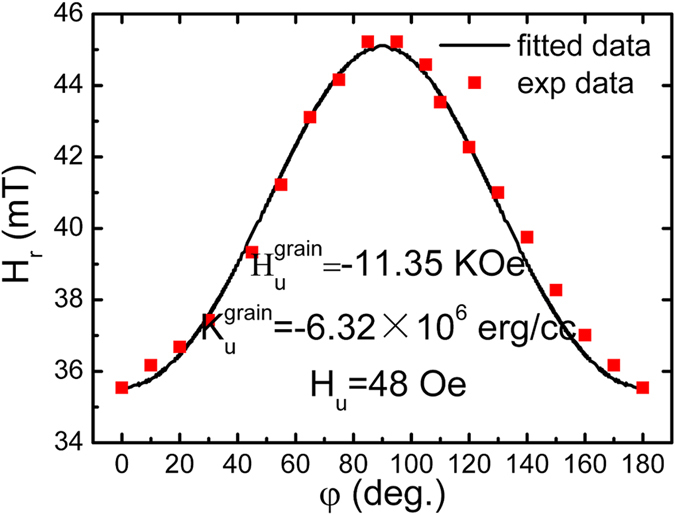
Resonance field as a function of the angle between the applied magnetic field and the easy axis for Co_81_Ir_19_ film with t = 50 nm. All the applied magnetic fields are parallel to the film plane during measurements.

To obtain an accurate value of the negative magnetocrystalline anisotropy energy, we performed an electron spin resonance (ESR) measurement of the Co_81_Ir_19_ film. The Co_81_Ir_19_ film with thickness of 50 nm is used because the orientation degree is the same in all of the samples with various thicknesses. Figure 4 shows the angle dependence of the resonance magnetic field, where φ is the angle between the applied magnetic field and easy axis of the film. Red square denotes the experimental data and the black line is the fitted curve, which matches well with each other. The key fitted magnetic parameters of Co_81_Ir_19_ film are 11.35 kOe, 48 Oe and −6.32 × 10^6^ erg/cc for the equivalent magnetocrystalline anisotropy field, in-plane uniaxial anisotropy field and magnetocrystalline anisotropy energy, respectively. The magnetocrystalline anisotropy energy is high, nearly equal to the demagnetizing energy 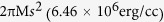. This result is relatively different from the conventional soft magnetic films with negligible magnetocrystalline anisotropy.

**Figure 5:**
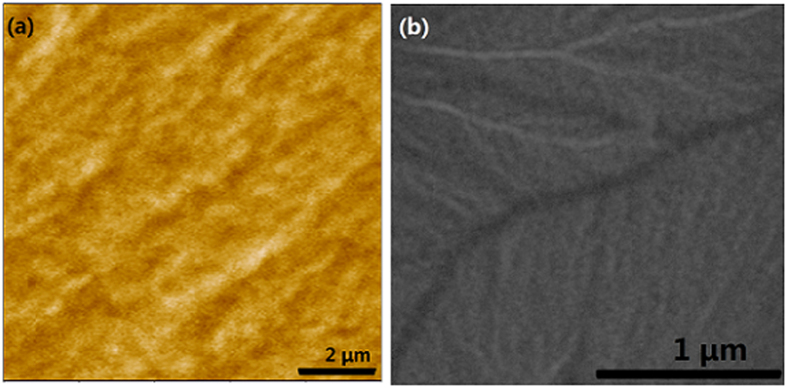
(**a**) MFM pattern of Co_81_Ir_19_ film with t = 50 nm. (**b**) The Lorentz TEM image of Co_81_Ir_19_ film with t = 50 nm.

MFM and Lorentz TEM technologies are used to characterize the domain structure and domain wall type for the oriented Co_81_Ir_19_ soft magnetic films under demagnetization state^26^. The measurement results for the sample with t = 50 nm are shown in Fig. 5. Figure 5a is the MFM image of the film. The image is uniform and no black or white line-shaped pattern across the image can be observed. As the MFM only detects the stray field perpendicular to the film plane, this result reveals that no Bloch wall is present in this film, and all magnetic moments lie in the film plane. To further validate the domain structure and domain wall form, the Lorentz TEM is performed on this sample and the result is shown in Fig. 5b. From this image, many black and white lines, which represent the domain walls in the film, can be observed. The film obviously has a multidomain structure. Considering that the domain wall exits in the film and the out-of-plane stray field cannot be detected through MFM, we can conclude that all of the magnetic moments lie in the film plane, and the domain wall is Néel wall. Note that, the Co_81_Ir_19_ layer thickness is 50 nm which is higher than that of the reported Fe-and Co-based soft magnetic films^18^,^19^,^20^,^21^,^22^.

**Figure 6:**
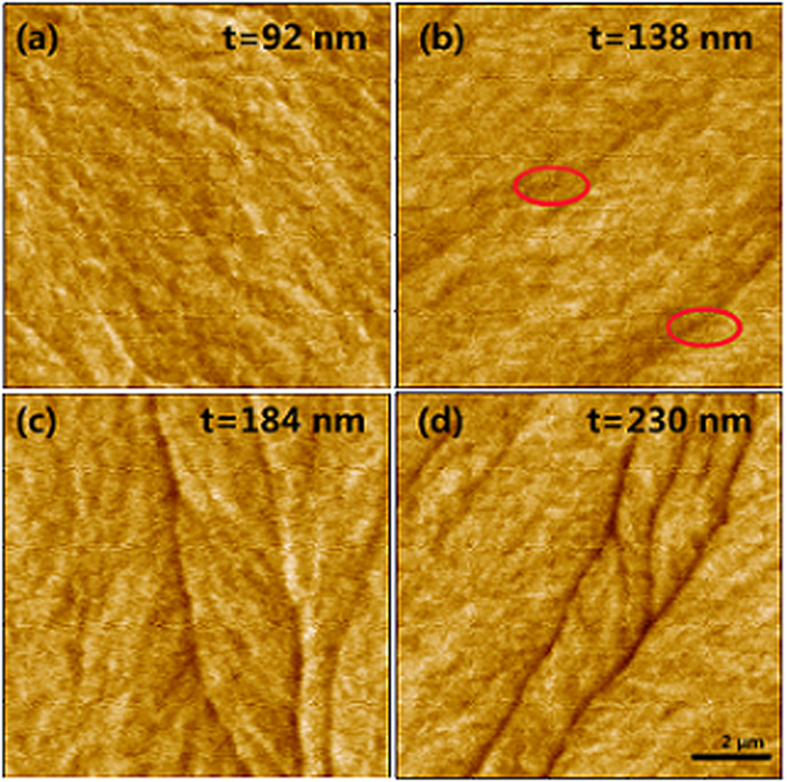
MFM pattern of Co_81_Ir_19_ films with t = 92 nm, 138 nm, 184 nm, 230 nm.

The observation in the thicker film is required to achieve the transition thickness from Néel wall to Bloch wall. As the Lorentz TEM image cannot be obtained on the thicker film because of the limited transmission depth of the electrons, only MFM technology is used for the thicker samples. Figure 6 shows the MFM images for the thicker samples with t = 92, 138, 184, and 230 nm under demagnetization state. When t = 92 nm, there is still no out-of-plane stray field, indicating the Néel wall survives to a film thickness of 92 nm. As the thickness increases to 138 nm, the black lines are observed in the MFM image, which demonstrates that the out-of-plane stray field appears in the film. This observation is a characteristic of Bloch wall. When the thickness increases to 184 and 230 nm, the line-shaped pattern remains unchanged, and the contrast becomes clearer. Compared with that in the MFM image at t = 184 and 230 nm, the line-shaped pattern in the MFM image for t = 138 nm is very weak; thus the transition thickness from Néel wall to Bloch wall of the oriented Co_81_Ir_19_ soft magnetic films is approximately 138 nm.

The theoretical transition thickness from Néel wall to Bloch wall of the soft magnetic Co_81_Ir_19_ film with strong magnetocrystalline anisotropy is investigated on the basis of eqs (1) and (2). The value of *K*_u_, 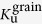, *M*_s_ and *A*_1_ is 2.43 ×10^4^ erg/cc, 6.32 × 10^6^ erg/cc, 1013.75 emu/cc and 1.0 × 10^−6^ erg/cm, respectively. Figure 6 indicates the calculated thickness dependence of 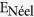 (black line) and 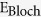 (red line). For comparison, the 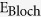 of the soft magnetic film with the same *K*_u_, *M*_s_ and *A*_1_ but without magnetocrystalline anisotropy is also calculated with the film thickness, and the result is shown in this figure as a blue line. When the magnetocyrstalline anisotropy is disregarded, the transition thickness from Néel wall to Bloch Wall is only 27 nm. This value is consistent with the reported value of the conventional soft magnetic film with negligible magnetocrystalline anisotropy. However, this transition thickness is enhanced to 130 nm when the strong magnetocrystalline anisotropy is introduced to the soft magnetic film. Our experimental result is in agreement with our calculated value. The high transition thickness from Néel wall to Bloch wall implies the magnetic moments can strictly lie in the film plane at a larger film thickness.

In summary, the magnetic domain structure and domain walls of the soft magnetic film with strong magnetocrystalline ansiotropy were investigated in experiment. Experimental and theoretical results show that the transition thickness from Néel wall to Bloch wall is dramatically enhanced compared with that of the conventional soft magnetic films with negligible magnetocrystalline anisotropy. This means that the Néel wall can exist in a thicker single-layer soft magnetic film, namely the magnetic moments can entirely lie in the film plane in a thicker single-layer soft magnetic film. This study proposes an important method to enhance the film thickness with persisting Néel wall in soft magnetic films by changing intrinsic magnetic parameters.

## Methods

### Fabrication of the oriented Co_81_Ir_19_ films

Ti, Au, and Co targets, and Ir square pieces were used to fabricate thin film by magnetron sputtering. The layer structure of the samples was substrate/Ti(9 nm)/Ru(13 nm)/Co_81_Ir_19_(t nm). The thickness of Co_81_Ir_19_ layer is 50 nm, 92 nm, 138 nm, 184 nm, and 230 nm. The silicon wafers with surface oxidation are used as substrate. Additionally, another film with Co_81_Ir_19_ layer of 50 nm was directly sputtered on microgrid for TEM and Lorentz microscope observation. The base pressure of vacuum was smaller than 3.0 × 10^−5^ Pa, and the sputter pressure was 0.25 Pa and 0.3 Pa for seed and CoIr layers, respectively.

### Characterization

Film composition was determined using an energy dispersive spectrometer (EDS). The X-ray diffraction technique (XRD) was applied to characterize the crystalline structure. Atomic force microscopy (AFM) and high resolution transmission electron microscopy (HRTEM) were performed to characterize the morphological feature and microstructure of the sample. The static magnetic properties were characterized using a vibrating sample magnetometer (VSM). The electron spin resonance (ESR) was used to characterize the in-plane and out-of-plane magnetocrystalline anisotropy fields. The magnetic domain structure of the sample was detected through magnetic force microscopy (MFM) and Lorentz transmission electron microscopy (LTEM) in a demagnetization state.

## Additional Information

**How to cite this article**: Xu, F. *et al.* Enhanced film thickness for Néel wall in soft magnetic film by introducing strong magnetocrystalline anisotropy. *Sci. Rep.* **6**, 20140; doi: 10.1038/srep20140 (2016).

## Figures and Tables

**Figure 7:**
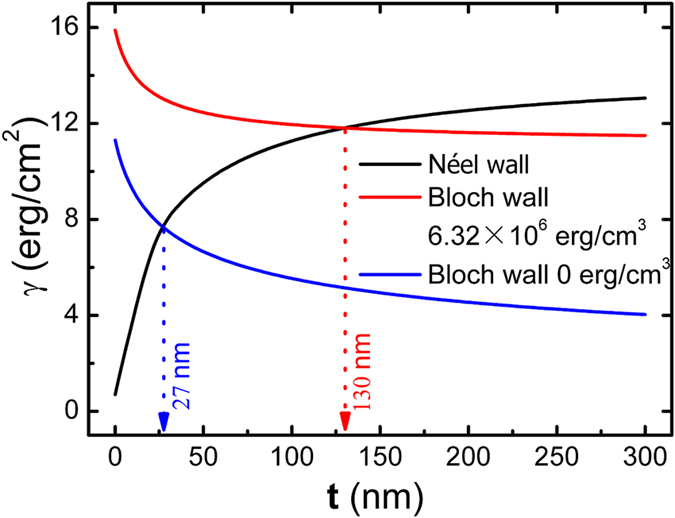
Co_81_Ir_19_ layer thickness dependence of domain wall energy for Néel and Bloch walls.
